# DEVELOPMENT OF AN INTERNATIONAL GERONTOLOGY EDUCATION COURSE BETWEEN THE US AND JAPAN

**DOI:** 10.1093/geroni/igad104.1093

**Published:** 2023-12-21

**Authors:** Takashi Yamashita, Flavius Lilly, Mio Kamijo

**Affiliations:** University of Maryland, Baltimore County, Baltimore, Maryland, United States; University of Maryland, Baltimore, Baltimore, Maryland, United States; University of Maryland, Baltimore, Baltimore, Maryland, United States

## Abstract

Sociocultural aspects of gerontology education have received growing attention in recent decades, as globalization and population aging resulted in more diverse older populations across global communities. Accordingly, gerontologists must equip with cultural and international competencies for their future careers, regardless of the fields, such as health care, long-term care, research, and education. This presentation outlines a development and case study (2023) of an international gerontology education course designed for graduate students in the U.S. After the preparation sessions, students take a faculty-led two-week trip to Japan, which is known as a super-aging society with nearly 30% of the population is age 65 years and older, and visit multiple aging-related sites including government agencies, research and education organizations, local communities, industries (e.g., robotics) and cutting-edge programs (e.g., health care research incubation program). The presentation offers three components. First, the academic objectives, such as the enhancement of cultural competencies and cross-national perspectives in gerontology, are described. Second, the practical course development process, including curriculum construction, planning trips, professional networking, student recruitment, funding source, coordination between two countries, and outcome assessment strategies, are illustrated. Finally, using the course evaluation data (n = 13 graduate students from 9 aging-related programs) and qualitative data (journals and field notes), positive outcomes (e.g., increased interest in working with socio-culturally diverse older adults) and challenges (e.g., funding international travel; scheduling difficulty; language barrier) are evaluated.

